# Soy protein isolate-sodium alginate colloidal particles for improving the stability of high internal phase Pickering emulsions: Effects of mass ratios

**DOI:** 10.1016/j.fochx.2023.101094

**Published:** 2023-12-27

**Authors:** Zhi Wang, Yubo Zhao, Haotian Liu, Qian Chen, Qian Liu, Baohua Kong

**Affiliations:** College of Food Science, Northeast Agricultural University, Harbin, Heilongjiang 150030, China

**Keywords:** High internal phase Pickering emulsion, Soy protein isolate, Sodium alginate, Emulsifying capacity

## Abstract

The potential of sodium alginate (SA) at different mass ratios to improve the emulsifying ability of soy protein isolate (SPI) in high internal phase Pickering emulsions (HIPPEs) was evaluated in this work. SPI-SA particles were used as a natural particle stabilizer of HIPPEs with 80 % oil phase. The properties of particles with varying SPI to SA ratios (10:0, 10:1, 10:3, 10:5, 10:10, and 10:15 w/w) were evaluated. HIPPEs with a 10:10 SPI to SA ratio exhibited the smallest droplet sizes. Both the storage modulus and loss modulus of the HIPPEs increased with increasing SA addition ratios, implying that HIPPEs with higher SA addition have stronger gel characteristics. In addition, super-resolution microscopy and cryogenic scanning electron microscopy indicated that SA addition strengthened the compactness of the interface film and increased the distribution uniformity of HIPPEs. In conclusion, the combination of SPI and SA is beneficial for improving the performance of HIPPEs.

## Highlights

• The performance of SPI-SA particles is superior to that of SPI particles.
• SPI-SA colloidal particles were used as stabilizers of HIPPEs with 80% oil phase.
• SPI-SA could form thick and dense interface layers on the surface of oil droplets.
• HIPPEs showed favorable rheological properties and stability.

## 1 Introduction

Pickering emulsions are stabilized by colloidal particles that are partially wetted by water and oil, rather than traditional emulsifier molecules. The main advantages of Pickering emulsions over conventional emulsions include the absence of surfactants, inhibition of droplet aggregation, and high stability (Ge et al., 2017). Pickering emulsions are classified into different types according to their internal dispersed phase volume fraction. With a minimum internal dispersion phase of 75 %, high internal phase Pickering emulsions (HIPPEs) have a distinctive gel network structure and texture that brings a rich flavor and a multi-layered sensory experience to food products, resulting in a wider range of applications (Huang, Lin, Zhang, & Tang, 2022). They are widely used in fat substitutes, dairy products, and 3D-printed food products (Galvão et al., 2022, Shi et al., 2020, Zhang et al., 2023). Wettability, surface charge, and Pickering particle size greatly influenced the formation of HIPPEs. Higher internal phase ratios endow HIPPEs with novel functional attributes different from those of conventional emulsions, such as a larger two-phase interface area and semi-solid gel rheological properties. Currently, many inorganic particles (*e.g.*, silica particles and graphene oxide) have been proven to be effective HIPPEs stabilizers (Gao et al., 2023), but their toxicity risk limits their application. Therefore, the creation of food-grade emulsifiers is very attractive to food researchers. Some biopolymer particles (*e.g.*, proteins and polysaccharides) have been reported to be good emulsifiers for HIPPEs (Liu et al., 2021, Liu et al., 2021). However, colloidal particles prepared with only proteins or polysaccharides exhibit poor emulsification ability. Hence, the preparation of composite particles by combining proteins with polysaccharides may be useful for stabilizing HIPPEs (Li, Xiong, Wang, Zhang, & Luo, 2023).

Soy protein isolate (SPI) is mainly composed of 7S and 11S globulins (Sui & Zhang, 2021). SPI is often used as a natural emulsifier because of its wide availability, low price, and high emulsifying properties (Wen, Zhang, Jin, Sui, & Jiang, 2020). Pickering emulsions can be prepared from soy protein particles without the addition of chemical modifications. However, external conditions such as pH, temperature, or ionic strength can affect the solubility of soy protein particles and their stable behaviors in Pickering emulsions (Li & de Vries, 2018). Wen et al. (2020) reported that SPI were unable to stabilize HIPPEs with 80 % oil volume fraction at neutral pH. Various treatments, such as heating, ultrasonication, and enzymatic digestion, have been used to improve the emulsification of SPI (Sui et al., 2021). Furthermore, the combination of SPI and polysaccharides is considered to be a promising strategy for improving emulsification. Liu et al., 2021, Liu et al., 2021 reported that bacterial cellulose nanofiber-SPI composite particles as emulsifiers can stabilize HIPPEs with a 75 % oil phase ratio. The properties of the composite particles were better than those of SPI particles, and HIPPEs prepared from the composite particles showed higher stability over a storage period of 2 months.

Hydrophilic polysaccharides are commonly used in the aqueous phase as thickening, gelling, or stabilizing agents to control the rheology of the continuous phase. Sodium alginate (SA), an anionic linear polysaccharide, can be dissolved in water (Thiviya et al., 2023). Pongsawatmanit, Harnsilawat, and McClements (2006) proved that β-lactoglobulin interacts with SA to form complexes that can stabilize emulsions. The pH of the system and the additive amount of SA determine the characteristics of the emulsions. There are few studies on using SA as a stabilizer for HIPPEs, while the properties of HIPPEs stabilized by SPI-SA complexes have not been investigated. Therefore, the effect of the SPI to SA ratio on the characterization of HIPPEs remains to be elucidated.

This work aims to develop HIPPEs stabilized by SPI-SA colloidal particles. The particle size, zeta-potential, and contact angle of SPI-SA colloidal particles with different SPI to SA ratios (10:0, 10:1, 10:3, 10:5, 10:10, and 10:15 w/w) were investigated and colloidal particle interactions were detected by Fourier transform infrared spectroscopy (FTIR) in this context. The physical performance and microstructure of HIPPEs stabilized with SPI-SA colloidal particles were characterized by analyzing the droplet size distribution, microscope images, and stability of HIPPEs.

## 2 Materials and methods

### 2.1 Materials

Soy protein isolate (SPI, protein ≥ 90 %) was supplied by Shandong Yuwang Ecological Food Co., Ltd. (Yucheng, China). Sodium alginate (SA, purity ≥ 98 %) was obtained from Yuanye Biotechnology Co., Ltd. (Shanghai, China). Sunflower oil was purchased from Jiusan Food Co., Ltd. (Harbin, China). All chemicals and reagents were of analytical grade.

### 2.2 Preparation of composite colloidal particles

The colloidal particles used in this study were made according to the method described by Yang et al., 2020, Peng et al., 2020 with some modifications. SPI was dispersed in distilled water (20 mg/mL) under stirring at 25 °C for 2 h and then stored at 4 °C overnight to allow the proteins to fully hydrate. Sodium azide (2 mM) was added to the SPI solution to limit the growth of microorganisms. SA (2, 6, 10, 20, and 30 mg/mL) was then dissolved in distilled water and stirred using a magnetic stirrer for 12 h to allow hydration. Subsequently, an equal volume of SPI solution was added to the SA solution, and mixtures with different SPI to SA ratios (10:1, 10:3, 10:5, 10:10, and 10:15 w/w) were obtained. The 10 mg/mL SPI water solution was used as a control (SPI:SA = 10:0). The mixtures were incubated in a water bath at 95 °C for 30 min to induce thermal crosslinking of SPI and SA. Finally, the above dispersions were sheared with a homogenizer at 15000 rpm for 2 min to obtain SPI-SA colloidal particles.

### 2.3 Particle size and zeta-potential of colloidal particles

The particle size and zeta-potential of the colloidal particles were measured with a Zetasizer Nano ZS90 (Malvern Instruments Ltd., Malvern, Worcestershire, UK) following the method described by Wang et al., 2023, Zhang et al., 2023. Before measurement, the samples were diluted 100 times using distilled water to avoid multiple light scattering. The refractive index of distilled water was set at 1.33. The particle size was reported as *Z*-average diameter (*D*_z_).

### 2.4 FTIR analysis of colloidal particles

The SPI-SA colloidal particle suspension was freeze-dried. The dried colloidal particle powders were then mixed with potassium bromide. FTIR of the colloidal particles with a wavenumber range of 4000–400 cm^−1^ was measured with a Nicolet iS50 FTIR instrument (Thermo Fisher Scientific, China) (Gao et al., 2023).

### 2.5 Three-phase contact angle of colloidal particles

According to the method of Meng et al. (2022), lyophilized SPI-SA colloidal particles were compressed into tablets under the pressure of 20 MPa. The tablets were placed in a quartz cuvette containing sunflower oil, then a high-precision syringe was extended into the cuvette and a 2-μL drop of water was placed on the tablets. Once the water droplets were stabilized, images were taken with the instrument’s camera (DataPhysics, GmbH, Berlin, Germany).

### 2.6 Preparation of HIPPEs

HIPPEs were prepared by homogenization and shearing following the method of Sun, Zhong, Zhao ,Li, Qi, and Jiang(2022). Briefly, the SPI-SA colloidal particles were homogenized using an Ultra-Turrax homogenizer (IKA T20 Basic, Staufen, Germany) at 10,000 rpm for 1 min, and sunflower oil was then added until the volume fraction of the oil phase reached 80 % with continuous high-shear mixing at 13,000 rpm for 3 min using a homogenizer. HIPPE is defined as an emulsion with a dispersed phase more than 74 %, and that 80 % of the oil phase used in this experiment meets this requirement. Because these four ratios of SPI to SA (10:1, 10:3, 10:5, 10:10) were successful in preparing HIPPEs, so, they were used in the following test.

### 2.7 Droplet size and distribution of HIPPEs

The droplet size and distribution of HIPPEs were determined using a Microtrac S3500 particle size analyzer (Microtrac Inc., PA, USA) (Sun et al., 2022). The samples were diluted 100 times with distilled water, then dripped into the feed port of the particle size analyzer, and the measurement was started when an appropriate absorption value was indicated by the instrument. The refractive indices of sunflower oil and distilled water were set at 1.47 and 1.33, respectively. The mean droplet diameter was represented as the volume-weighted mean diameter (*D*_4,3_).

### 2.8 Microstructure of HIPPEs

#### 2.8.1 Optical microscope observation

The microstructure of HIPPEs was examined with an optical microscope (Olympus, UIS2, Japan). A small amount of HIPPE was placed on a microscope slide. The coverslip was gently placed over the HIPPE, and then the samples were observed under a 100 × objective lens (Galvão et al., 2022).

#### 2.8.2 Cryogenic scanning electron microscopy observation

Cryogenic scanning electron microscopy (cryo-SEM) observation of the HIPPEs was carried out with an S-3400 N scanning electron microscope equipped (Hitachi, Japan) following the method of Xiao, Wang, Gonzalez, and Huang (2016). A drop of the sample was placed on the aluminum holder and frozen using boiling liquid nitrogen in the slush station before transferring it to the preparation chamber at –160 °C under vacuum, which was fitted with a blade to fracture the sample. After fracturing, the sample was transported to the cryo-transfer equipment and sublimated at –100 °C for 15 min before being coated with gold. The coated samples were placed in an observation chamber with an SEM stage cold module set to –140 °C.

#### 2.8.3 Super-resolution microscopy observation

Super-resolution microscopy images of HIPPEs were captured using the DeltaVision OMX SR imaging system (General Electric Company, Healthcare, Massachusetts, US). HIPPEs were stained with 0.1 % Nile Red staining solution and 0.1 % Nile Blue staining solution. Stained HIPPEs were then placed on the slides to observe the droplet distribution and interface structure. The excited wavelengths of Nile blue and Nile red were 642 nm and 488 nm, respectively. The samples were examined using the 60 × /1.42NA PlanApo oil immersion lens (Olympus Co., Tokyo, Japan) and the scan model was set to conventional wide-field imaging.

### 2.9 Stability tests of HIPPEs

#### 2.9.1 Centrifugal stability of HIPPEs

The stability of the HIPPEs was determined by centrifugation according to the method of Li et al. (2023). Five grams of samples were placed in a tube and then centrifuged at 10,000 *g* for 10 min at 4 °C. The appearance of the samples was recorded with a digital camera.

#### 2.9.2 Heat stability of HIPPEs

The HIPPEs were placed in sample bottles and heated at 75 °C for 30 min. Photographs were taken to document the appearance of the HIPPE samples both before and after they were subjected to heat. The microstructure of HIPPEs before and after heating was examined with an optical microscope (Olympus, UIS2, Japan) according to the above method shown in section 2.8.1. The heating condition of 75 °C for 30 min was selected is because that HIPPEs will be used in emulsified sausages as a fat substitute in our further study, and this heating condition can be used in emulsified sausage processing.

#### 2.9.3 Freeze-thaw stability of HIPPEs

The HIPPEs were placed in sample bottles and frozen at –20 °C for 24 h. The samples were then thawed at room temperature, and the appearance of the samples was recorded by taking photographs.

#### 2.9.4 Storage stability of HIPPEs

The HIPPEs were placed in sample bottles and stored at 4 °C for 14 days. Photographs were taken to document the appearance of the HIPPE samples. The microstructure of HIPPEs at 0 and 14 days was examined with an optical microscope (Olympus, UIS2, Japan) according to the above method shown in section 2.8.1.

### 2.10 Rheological behavior of HIPPEs

The rheological properties of the HIPPEs were determined using a dynamic shear rheometer (DHR-1 rheometer, TA Instruments, Delaware, USA) equipped with a parallel plate (1.0 mm gap, 40 mm diameter) (Gao et al., 2023). The moduli of the HIPPEs were recorded in the frequency scan mode in 10–100 rad/s range. The viscosity of the HIPPEs was measured with shear rates increasing from 10 to 100 s^−1^. Measurements were performed at a constant strain amplitude of 1 % (in the linear viscoelastic region). All measurements were conducted at 25 °C.

### 2.11 Statistical analysis

Three separate sample batches were prepared. For each batch, the relevant traits were measured in triplicate. Statistical calculations were conducted using a statistical software package (Statistix 8.1, Analytical Software, Minnesota, USA) and represented as means ± standard error (SE). Significance between means (*P* < 0.05) was analyzed by Tukey’s multiple comparisons test.

## 3 Results and discussion

### 3.1 Particle size and zeta-potential of colloidal particles

**Fig. 1:**
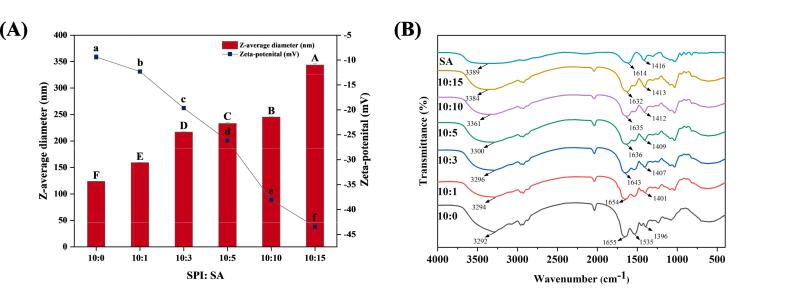
Particle size, zeta-potential (A) and Fourier transform infrared spectroscopy (B) of SPI-SA colloidal particles with different ratios (SPI: SA = 10:0, 10:1, 10:3, 10:5, 10:10 and 10:15). Letters A–F and a-f indicate significant differences in particle size and zeta-potential, respectively, between different treatments (*P* < 0.05).

The strength of the interaction forces between colloidal particles can be reflected by the particle size (Gao et al., 2023). As shown in Fig. 1**A**, all colloidal particles were between 126 and 352 nm, indicating that the SPI-SA composite was nano-sized. The particle sizes of the SPI-SA colloidal particles were larger than those of SPI without SA (*P* < 0.05) and significantly increased with increasing SA addition ratios (*P* < 0.05), which might be due to electrostatic interactions, hydrophobic interactions, and hydrogen bonding between SPI and SA (Zhou et al., 2022). The observed SA level-dependent particle size behavior was very similar to that mentioned by Jin, Zhu, Jiang, Shao, Li, and Huang (2017). For Pickering emulsions, larger particles have a greater contact area with the oil–water interface, and thus the desorption energy is higher (Gao et al., 2023). As a result, larger particle sizes are advantageous for Pickering emulsion stabilization. Nevertheless, oversized particles are detrimental to Pickering emulsion formation (Ge et al., 2017).

The zeta-potential values of the colloidal particles were all negative as presented in Fig. 1**A**. With increasing SA addition ratios, the absolute magnitude of the zeta-potential increased (*P* < 0.05). The net charge of the systems was strongly dependent on the SA addition ratios and the SPI-SA colloidal particle with a 10:15 ratio had the highest net charge (–43.48 mV). SA had a large amount of carboxyl groups, which are negatively charged because they were ionized in solution (Wu, et al., 2018). With the increase in SA addition ratio, the content of carboxyl groups in the system increases, so the absolute value of negative charge increases. In general, the higher the absolute value of the zeta-potential, the greater the electrostatic repulsion between the particles, the less likely that the particles are to bind to each other, and the more stable the system is (Wu, et al., 2018). The zeta potential of the SPI-SA colloidal particles was higher than that of SPI alone, indicating that the repulsive electrostatic interaction was enhanced by the addition of SA. The above consequence agrees with the former work of Zhang, Fu, et al. (2023), which showed that the addition of SA to an eggshell particle suspension led to an increase in the absolute value of the potential, thus enhancing the stability of the system.

### 3.2 FTIR analysis

FTIR analysis of the SPI-SA colloidal particles was performed to investigate the molecular interactions between SPI and SA. As shown in Fig. 1**B**, the spectra of all samples demonstrate a broad peak at 3700–3200 cm^−1^ (amide A), which can be attributed to the intermolecular H-bonded N—H and O—H stretching vibrations (Luo, Zhang, Whent, Yu, & Wang, 2011). The amide A peak in SPI moved to higher wavenumbers with increasing SA level, suggesting that hydrogen bonds were formed between the amide groups in SPI and the carboxyl or hydroxyl groups in SA (Başyiğit, Altun, Yücetepe, Karaaslan, & Karaaslan, 2023). SA exhibited characteristic absorption peaks at 1614 and 1416 cm^−1^, which were attributed to asymmetric ​and symmetric COO − stretching vibration. The COO − symmetric stretching vibrations of SA shifted to a higher wavenumber with increasing SA addition ratios, indicating that hydrogen bonds were formed between SPI and SA (Siddaramaiah, Swamy, Ramaraj, & Lee, 2008). The spectra of SPI show characteristic absorption peaks at 1655 cm^−1^ (amide I) and 1535 cm^−1^ (amide Ⅱ) corresponding to the C=O stretching vibration and N — H bending vibration, which agrees well with previous studies (Zhou et al., 2022). The amide I peak of SPI shifted to a lower wavenumber as the SA content increased, indicating the variation of the secondary structure of SPI, as well as the electrostatic interactions, which occurred between SPI and SA (Fan, Yuan, Guo, & Kang, 2022). While the amide Ⅱ peak intensity of SPI decreased with increasing SA content, suggesting that hydrophobic interactions occurred between SPI and SA (Wang, Sun, Xu, Du, Zhu, & Wu, 2021). These interactions may determine the emulsification and wettability of SPI-SA colloidal particles.

### 3.3 Three-phase contact angle of colloidal particles

**Fig. 2:**
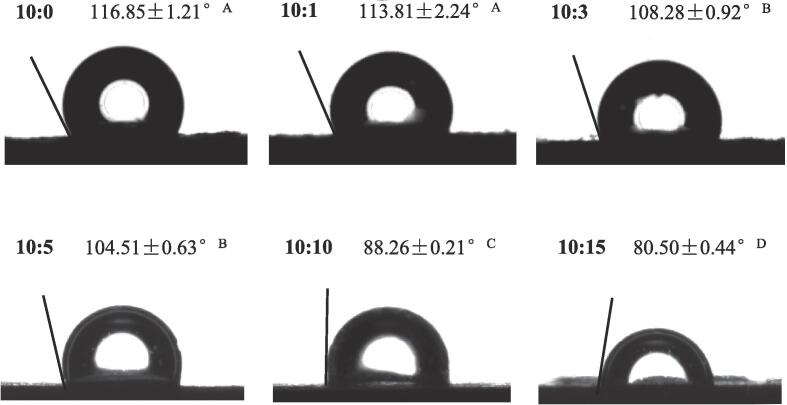
Three-phase contact angle of SPI and SPI-SA colloidal particles with different ratios (SPI: SA = 10:0, 10:1, 10:3, 10:5, 10:10 and 10:15). Letters (A-D) indicate significant differences between the different treatments (*P* < 0.05).

Proper wettability of colloidal particles is vital for forming the stabilized Pickering emulsion (Ge et al., 2017). Particle wettability can be determined by measuring the three-phase contact angle to assess the emulsifying capacity of solid particles. Normally, the closer the particle contact angle is to 90°, the higher the particle desorption energy, which means that the particles can protect droplets from aggregation (Li et al., 2023). As shown in Fig. 2, the contact angle values of SPI-SA colloidal particles were lower than that of SPI alone (*P* < 0.05), which revealed that the surface of excessively hydrophobic SPI particles can be successfully modified by hydrophilic SA. In addition, the higher contact angle of the SPI alone colloidal particles (116.85°) was probably due to the hydrophobic amino acid residues in the soy protein molecule (Liu et al., 2021, Liu et al., 2021). With increasing SA addition ratios, the contact angle value of the composite particles decreased significantly (*P* < 0.05). The three-phase contact angle value of SPI-SA colloidal particles with a 10:10 ratio was 88.26°, which was the closest to 90°. Therefore, SPI-SA colloidal particles with a 10:10 ratio exhibits the highest emulsification ability among all samples. The results revealed that the composite colloidal particles had higher emulsification ability compared to pure SPI because of their proximity to the 90° contact angle. These findings are in agreement with the study of Su et al. (2021), who discovered that the addition of propylene glycol alginate to β-lactoglobulin significantly reduced the contact angle of the particles, and the decreased degree was closely related to the protein to polysaccharide mass ratio. Furthermore, SA has hydrophilic hydroxyl groups, which may cause a decrease in the contact angle of the composite particles.

### 3.4 Appearance, droplet size, and droplet size distribution of HIPPEs

**Fig. 3:**
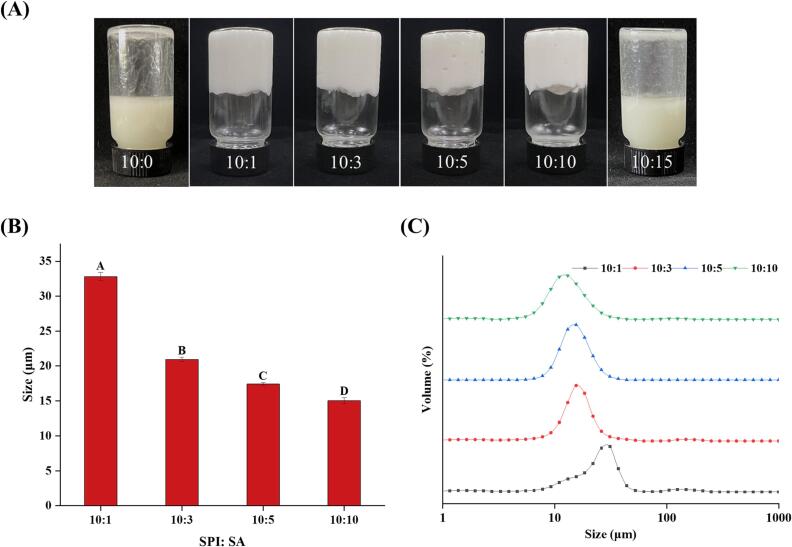
Appearance (A), droplet size (B) and droplet size distribution (C) of HIPPEs stabilized by SPI-SA colloidal particles with different ratios (SPI: SA = 10:1, 10:3, 10:5 and 10:10). Letters (A-D) indicate significant differences in droplet size between the different treatments (*P* < 0.05).

The impact of the SPI to SA ratio on the appearances of bottled HIPPEs formed from SPI-SA colloidal particles is shown in Fig. 3**A**. Emulsions formed from colloidal particles with 10:0 and 10:15 SPI to SA ratios were unstable. Conversely, when the SPI to SA ratios were 10:1, 10:3, 10:5, and 10:10, the formed HIPPEs were relatively non-flowing when the container was inverted, suggesting that SPI-SA had great potential for the manufacture of gelatinous HIPPEs at these ratios. Similarly, Huang et al., (2022) detected that polysaccharides can facilitate the development of gel networks in the HIPPEs produced by heated SPI. The appearance of the bottled HIPPEs had no significant changes as the SA addition ratios increased from 10:1 to 10:10. An unstable emulsion was developed from the colloidal particles at a 10:0 SPI to SA ratio, which may be because there was high hydrophobicity in the 10:0 ratio samples because of the high SPI content (Zhao, Cui, Ma, McClements, Liu, & Liu, 2022). While the SPI-SA particles at a 10:15 SPI to SA ratio were also unable to form stable HIPPEs, probably because the excess SA made the particles too hydrophilic and the excess polysaccharide pumped the protein off from the surface of the oil droplets (Wang et al., 2022). This may be caused by a large aggregation, driven by hydrophobic interaction and electrostatic attraction and formed by SPI and SA due to the high concentration of SA. Because the 10:0 and 10:15 SPI to SA ratios cannot form stable HIPPEs, they were not used in the following analysis.

Emulsions with smaller droplets have higher stability (Shi, Feng, Wang, & Adhikari, 2020). Fig. 3B and C showed the droplet size and distribution of the four HIPPEs treatments (SPI:SA = 10:1, 10:3, 10:5, and 10:10). With increasing SA addition ratios, the average droplet size significantly decreased (*P* < 0.05). Oil droplet sizes decreased progressively (*P* < 0.05) with increasing SA addition ratios in colloidal particles, and the 10:10 SPI to SA ratio had the smallest droplet sizes. The droplet size distribution followed similar trends with droplet sizes. All HIPPEs samples exhibited a narrow peak of droplet size distribution. With increasing SA addition ratios, the center of the peak shifted significantly to the left. Li et al., (2023) also demonstrated that pectin reduced the oil droplet size of emulsions and thickened the emulsion system. These results indicated that the addition of appropriate polysaccharides (SA) can reduce the droplet size of HIPPEs.

### 3.5 Microscopic observation of HIPPEs

#### 3.5.1 Optical microscope

**Fig. 4:**
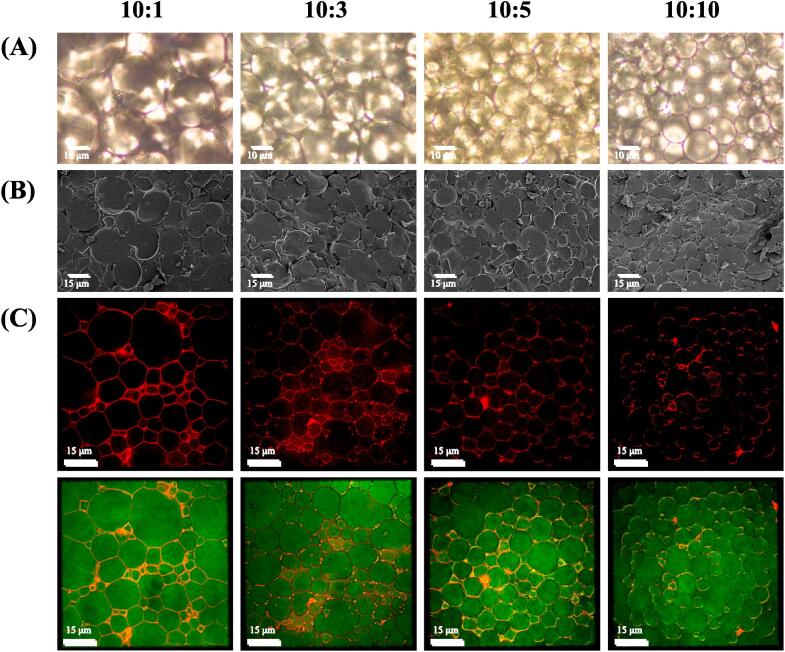
Optical microscope images (A), cryogenic scanning electron microscopy (cryo-SEM) images (B) and super-resolution microscopic images (C) of HIPPEs stabilized by SPI-SA colloidal particles with different ratios (SPI:SA = 10:1, 10:3, 10:5 and 10:10).

The droplet size and distribution of the HIPPEs were observed under the microscope (Fig. 4). The images show that the HIPPEs were successfully prepared and there was no phase separation (Fig. 4**A**). HIPPEs droplets with a 10:1 SPI to SA ratio exhibited larger size and more heterogeneous spherical shape than those of other samples. With increasing SA addition ratios, the droplets became smaller and presented a more homogeneous state. It can be observed that all the HIPPEs samples are tightly packed with droplets forming a dense gel network and two adjacent droplets share an interfacial layer, which is in accordance with the findings of Peng et al., 2020, Yang et al., 2020. This suggests that SA favored the formation of stable HIPPEs, which is consistent with the droplet size distribution findings.

#### 3.5.2 Cryogenic scanning electron microscopy

Cryo-SEM, an emerging technique, can be applied to characterize the distributions of SPI-SA colloidal particles and sunflower oil in HIPPEs. As shown in Fig. 4**B**, the oil droplets were densely packed with most of them enclosed in a network produced by SPI-SA colloidal particles, and the network can act as a three-dimensional barrier (Sun et al., 2022). The colloidal particles can bind adjacent droplets and create a strong stabilizing effect across the spatial barrier. With increasing SA ratio, the oil droplet size decreased and the oil droplet dispersion became more uniform. Thus, composite particles prepared with a 10:10 SPI to SA ratio are more suitable for stabilizing HIPPEs. Guo, Wu, Du, Lin, Xu, and Yu (2021) observed the HIPPEs prepared with casein by cryo-SEM and found the droplets of HIPPEs were tightly aggregated.

#### 3.5.3 Super-resolution microscopy

Fig. 4**C** presents super-resolution microscopy pictures of HIPPEs with different SPI to SA ratios. The sunflower oil appears green, while the colloidal particle appears red. The green polygonal droplets were wrapped in a dense layer of red fluorescence, demonstrating that the type of all HIPPEs were oil in water. Moreover, the protein-polysaccharide particles were either on the surface of the oil droplet or within the surrounding aqueous phase. The differences in droplet size among all the HIPPEs samples were intuitive. These results show that HIPPEs are stabilized by colloidal particles adsorbed on the droplet surface and that the oil is well encapsulated by the colloidal particles, which are similar to those of HIPPEs prepared by gelatin (Tan, Zhao, Tian, Wen, Gou, & Ngai, 2017). At the relatively low SA addition ratios, the HIPPEs contained large polygonal oil droplets tightly packed together. As the SA addition ratios increased, the green droplets became progressively smaller and more homogeneous. The above results were similar to those of droplet size, optical microscopy, and cryo-SEM results.

### 3.6 Stability tests

#### 3.6.1 Centrifugal stability of HIPPEs

**Fig. 5:**
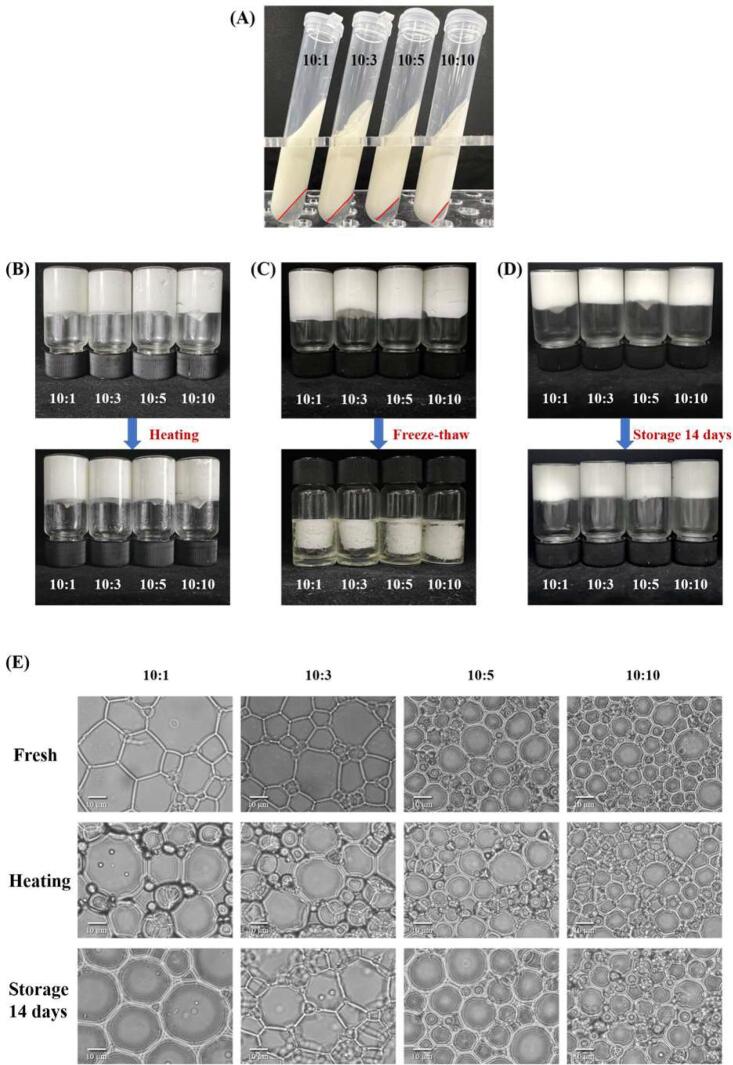
Appearance of HIPPEs stabilized by SPI-SA colloidal particles with different ratios (SPI:SA = 10:1, 10:3, 10:5 and 10:10) after centrifugation (A), heating (B), freeze–thaw (C), and storage (D). Optical microscope (E) of HIPPEs stabilized by SPI-SA colloidal particles with different ratios (SPI:SA = 10:1, 10:3, 10:5 and 10:10) before and after heating and storage.

Centrifugal stability analysis provides valuable information on the stability of HIPPEs under mechanical forces, as well as being an important indicator of emulsion quality (Li et al., 2023). As shown in Fig. 5**A,** the emulsion morphology after centrifugation was clearly dependent on the SPI to SA ratios. All samples exhibited varying degrees of phase separation. Apparently, colloidal particle-stabilized HIPPEs with a 10:1 SPI to SA ratio were relatively unstable, and a higher water layer was observed after centrifugation. While the bottom water layer of the HIPPEs decreased significantly with increasing SA addition ratios. Samples with a 10:10 SPI to SA ratio were more stable, with almost no water layer in the lower layer after centrifugation. This may be because the droplets in this system are smaller and encapsulated by a dense layer composed of particles. This compact interfacial layer is formed by the network of colloidal particles in the continuous phase and the steric hindrance effect, which prevents the oil droplets from flocculating or coalescing (Xu et al., 2023). Zhang, Wu, Li, Li, Pei, and Liu (2022) also demonstrated that the stability of emulsions stabilized with bacterial cellulose nanofibrils-SPI composites was superior to that of SPI-stabilized emulsions, and their stability depended on the additive amount of bacterial cellulose nanofibrils in the complexes.

#### 3.6.2 Heat stability of HIPPEs

Heat treatment is one of the most dominant treatments in food processing, so it is necessary to assess the effect of heat treatment on HIPPEs with different SPI to SA ratios. As shown in the Fig. 5**B**, there was no significant change in the appearance of the HIPPEs before and after heating, indicating they have good thermal stability and can be used in food systems requiring heat treatment. The microscopic observation showed that the HIPPEs prepared by SPI-SA colloidal particles had a good thermal stability, retaining a stable structure even after heating (Fig. 5**E**). The droplet size of HIPPEs whether heated or not gradually decreased with increasing SA addition ratios. The same phenomenon has been observed in HIPPEs formed with black soybean isolate protein and cyanidin-3-O-glucoside (Cui et al., 2022).

#### 3.6.3 Freeze-thaw stability of HIPPEs

The apparent morphology of the freeze–thaw treatment on HIPPEs with different SPI to SA ratios is shown in Fig. 5**C**. Some oil and water leakage in all samples after freeze–thaw treatment, indicating that the emulsion was disrupted. There are two factors that likely contributed to the leakage of sunflower oil from the complex colloidal particles. Firstly, it is probable that the HIPPEs formed ice crystals during the freezing process, which disrupted their structure when melted (Zhang, Zhao, et al., 2023). Secondly, the freeze–thaw treatment may have disrupted the intra or intermolecular interaction forces of the proteins, altering their conformation and preventing them from maintaining interfacial stability (Zhang, Li, Xia, Sun, Sun, & Kong, 2023). As the percentage of SA increases, the freeze–thaw treated HIPPEs precipitate less oil phase and the freeze–thaw stability of the emulsion is improved. This phenomenon was due to the formation of a protective layer of macromolecules around the protein layer by SA, which reduced the likelihood of emulsion flocculation during freeze-thawing.

#### 3.6.4 Storage stability of HIPPEs

As shown in Fig. 5**D**, compared to 0 days, there were no obvious changes in appearance for all of the samples after 14 days of storage. All the HIPPE samples before and after storage had no flowing when the sample bottles were inverted. To further characterize the storage stability of HIPPEs, the microstructures of HIPPEs were observed with optical microscope (Fig. 5**E)**, which revealed that except for 10:1 of SPI to SA ratio, HIPPE droplet sizes of other three samples had no obvious changes. At the some storage time, the droplet size of HIPPEs gradually decreased with increasing SA addition ratios. These demonstrated that HIPPEs prepared by SPI-SA colloidal particles had a high storage stability.

### 3.7 Rheological behavior of HIPPEs

**Fig. 6:**
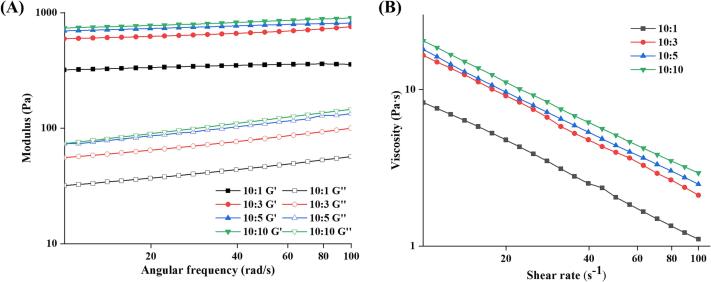
Rheological properties (A) and viscosity (B) of HIPPEs stabilized by SPI-SA colloidal particles with different ratios (SPI: SA = 10:1, 10:3, 10:5 and 10:10).

To investigate the potential pattern of SPI-SA in stabilizing HIPPEs, the modulus and apparent viscosity of the samples were measured (Fig. 6**A and 6B**). The energy storage modulus (*G'*) of a material reflects its capacity to store energy during deformation, whereas loss modulus (*G“*) shows its ability to lose energy (Zhang, Wang, Huang, & Xu, 2024). *G'* is greater than *G''* for all samples, indicating that HIPPEs tended to be more elastic or solid-like (Galvão et al., 2022). The *G'* of the HIPPEs significantly enhanced with the change of SPI to SA ratio from 10:1 to 10:10, implying that HIPPEs with higher SA addition have stronger gel characteristics, which is consistent with the results of centrifugal stability tests. Yan, McClements, Zhu, Zou, Zhou, and Liu (2019) also demonstrated that the rheological behavior of HIPPEs stabilized by octenyl succinic anhydride-modified starch and chitosan improved with increasing chitosan addition ratios. In addition, *G'* and *G''* exhibited a relatively weak frequency dependency, indicating the rheological properties of all samples were not strongly affected by the applied deformation, even at high frequencies (Zhang, Wang, Huang, & Xu, 2024).

The shear viscosity measurement results of the HIPPEs are shown in Fig. 6**B**. With increasing shear rate, the apparent shear viscosity of all samples decreased, indicating that all samples exhibited typical non-Newtonian pseudoplastic behavior (Li et al., 2023). At a shear rate of 10–100 s^−1^, the apparent viscosity increased with increasing SA addition ratios and was greatest at a 10:10 SPI to SA ratio, indicating that it formed a better gel-like network structure in this shear rate range (Sun et al., 2022). The inclusion of polysaccharides may have enhanced the structure of HIPPEs, as seen by the increase in apparent shear viscosity of HIPPEs with higher proportions of SA addition. This may be because the droplets of HIPPEs are smaller and denser with high SA addition ratios, resulting in low droplet mobility and enhanced resistance to deformation.

## 4 Conclusions

In this study, HIPPEs were fabricated using SPI-SA composite colloidal particles. The stability and wettability of the SPI-SA colloidal particles were enhanced with the addition of SA. FTIR revealed that hydrogen bonds, electrostatic interactions, and hydrophobic interactions played an important role in the formation of SPI-SA colloidal particles. Furthermore, the stability, rheological behavior, and interface network structure of the HIPPEs were enhanced with the increased SA addition ratio, and a 10:10 SPI to SA ratio was the optimal ratio of SPI-SA colloidal particles for stabilizing HIPPEs. This study suggests that SPI-SA colloidal particles have good application potential in the preparation of HIPPEs, which can be used as soft materials similar to solid fats, tissue engineering scaffolds, and slow-release delivery systems for bioactive substances.

## CRediT authorship contribution statement

**Zhi Wang:** Data curation, Visualization, Writing – original draft. **Yubo Zhao:** Investigation, Formal analysis. **Haotian Liu:** Conceptualization, Methodology. **Qian Chen:** Investigation, Formal analysis. **Qian Liu:** Investigation, Software. **Baohua Kong:** Supervision, Project administration, Funding acquisition.

## Declaration of competing interest

The authors declare that they have no known competing financial interests or personal relationships that could have appeared to influence the work reported in this paper.

## Data Availability

The data that has been used is confidential.
